# Mapping the situation of research on coronavirus disease-19 (COVID-19): a preliminary bibliometric analysis during the early stage of the outbreak

**DOI:** 10.1186/s12879-020-05293-z

**Published:** 2020-08-01

**Authors:** Sa’ed H. Zyoud, Samah W. Al-Jabi

**Affiliations:** 1 Poison Control and Drug Information Center (PCDIC), College of Medicine and Health Sciences, An-Najah National University, Nablus, 44839 Palestine; 2 Department of Clinical and Community Pharmacy, College of Medicine and Health Sciences, An-Najah National University, Nablus, 44839 Palestine; 3 Clinical Research Centre, An-Najah National University Hospital, Nablus, 44839 Palestine

**Keywords:** Bibliometric, Scopus, COVID-19, Novel coronavirus, 2019-nCoV

## Abstract

**Background:**

The novel coronavirus, named as 2019-nCoV or coronavirus disease 2019 (COVID-19), has recently appeared in China and has spread worldwide, presenting a health threat to the global community. Therefore, it is important to understand the global scientific output of COVID-19 research during the early stage of the outbreak. Thus, to track the current hotspots, and highlight future directions, we performed a bibliometric analysis to obtain an approximate scenario of COVID-19 to date.

**Methods:**

Relevant studies to COVID-19 were obtained from the Scopus database during the early stage of the outbreak. We then analysed the data by using well-established bibliometric indices: document type, country, collaboration patterns, affiliation, journal name, and citation patterns. VOSviewer was applied to map and determine hot topics in this field.

**Results:**

The bibliometric analysis indicated that there were 19,044 publications on Scopus published on COVID-19 during the early stage of the outbreak (December 2019 up until June 19, 2020). Of all these publications, 9140 (48.0%) were articles; 4192 (22.0%) were letters; 1797 (9.4%) were reviews; 1754 (9.2%) were editorials; 1728 (9.1%) were notes; and 433 (2.3%) were others. The USA published the largest number of publications on COVID-19 (4479; 23.4%), followed by China (3310; 17.4%), Italy, (2314; 12.2%), and the UK (1981; 10.4%). *British Medical Journal* was the most productive. The *Huazhong University of Science and Technology, Tongji Medical,* and *Harvard Medical School* were the institutions that published the largest number of COVID-19 research. The most prevalent topics of research in COVID-19 include “clinical features studies”, “pathological findings and therapeutic design”, “care facilities preparation and infection control”, and “maternal, perinatal and neonatal outcomes”.

**Conclusions:**

This bibliometric study may reflect rapidly emerging topics on COVID-19 research, where substantial research activity has already begun extensively during the early stage of the outbreak. The findings reported here shed new light on the major progress in the near future for hot topics on COVID-19 research including clinical features studies, pathological findings and therapeutic design, care facilities preparation and infection control, and maternal, perinatal and neonatal outcomes.

## Background

A cluster of viral pneumonia cases of unknown cause, subsequently identified as a novel coronavirus, named as 2019-nCoV or COVID-19, was detected on December 31, 2019, in Wuhan, China [1–4]. The disease has spread rapidly from Wuhan to other regions in China. Further, the dissemination of this virus has been observed in 216 countries and over 535,700 deaths as of 7 July 2020 [5].

The clinical symptoms of COVID-19 range from asymptomatic to severe pneumonia and multiple organ failure [6]. The most commonly reported clinical features are fever, cough, breathlessness, myalgia, and fatigue, whereas less common reported clinical features to include diarrhea, headache, conjunctivitis, and runny nose [7, 8]. For a subset of patients, the disease may progress to pneumonia with respiratory failure and even death by the end of the first week [8, 9]. At this time, there are few specific antiviral strategies combined with supportive treatment, but several potent nominees of antivirals such as lopinavir/ritonavir, remdesivir, or chloroquine and repurposed drugs are under urgent investigation [10].

Bibliometric evaluation, a commonly accepted statistical tool, helps to present knowledge structures of a particular research field [11–13]. Throughout recent years, bibliometrics have been used to provide strong insights into several biomedical fields linked to many virus outbreaks [14–27]. There have been a few recent reviews of COVID-19 or Coronavirus [28–36], but no comprehensive evaluation of the existing research on COVID-19 has yet been performed or published. The previously published bibliometric studies [28–36] on COVID-19 have been published by using PubMed or Web of Science (WoS) database for data collection and were limited to biomedical research areas. Therefore, the purposes of the current study were to assess the global scientific output of COVID-19 research during the early stage of the outbreak through bibliometric analysis, determine the top-cited publications, and to explore the current hot topics in order to provide the scientists and researchers with a resource that can help them by identifying the current research priorities.

## Methods

### Data source

Published papers were retrieved via a topic search (title/abstract) of the Scopus on 19 June 2020. In the current analysis, the Scopus database was used without restricting the findings to any particular field of search as a difference from previous bibliometric studies on COVID-19 [28–36]. The use of Scopus as a bibliometric resource in our study was based on the truth that it has the world’s largest abstract and citation database of peer-reviewed scientific literature compared with PubMed or Web of Science [37–39].

### Search strategy

Concerning COVID-19 during the early stage of the outbreak, the terms used in the search engine of Scopus were either in Title or Abstract (“COVID 19” or “2019 novel coronavirus” or “coronavirus 2019” or “coronavirus disease 2019” or “2019-novel CoV” or “2019 ncov” or COVID 2019 or COVID19 or “corona virus 2019” or nCoV-2019 or nCoV2019 or “nCoV 2019” or 2019-ncov or COVID-19 or “Severe acute respiratory syndrome coronavirus 2” or “SARS-CoV-2”).

### Bibliometric analysis

All relevant data to COVID-19 were downloaded from the Scopus. In this study, we analyzed the retrieved data through Excel to collect the following bibliometric indicators based on previous similar studies [40–43]: (1) publication output; (2) document type; (3) country/region; (4) institute; (5) journal; (6) *h*-index; and (7) citation.

### Visualized analysis

VOSviewer v.1.6.14 (https://www.vosviewer.com/) is frequently used to construct and visualize network terms used in title/abstract articles to detect hot topics in this field [44, 45]. The policy adopted by Scopus does not provide complete information on all the data and allows for the export of up to 2000 articles. The exported file is in an excel file format. Therefore, we decided to export the top 2000 cited articles and further analyzed them to construct and visualize networks terms used in title/abstract articles to detect hot topics in this field.

## Results

The bibliometric analysis indicated that there were 19,044 publications on Scopus published related to COVID-19 during the early stage of the outbreak (December 2019 up until June 19, 2020). Of all these publications, 9140 (48.0%) were articles; 4192 (22.0%) were letters; 1797 (9.4%) were reviews; 1754 (9.2%) were editorials; 1728 (9.1%) were notes; and 433 (2.3%) were others. In addition, the *h*-index for all data collected related to the research of COVID-19 was 108.

The publications linked to COVID-19 included authors from 159 different countries. The top 10 countries published 16,957 (89%) articles each are presented in Table 1. The USA published the largest number of publications on COVID-19 (4479; 23.4%), followed by China (3310; 17.4%), Italy, (2314; 12.2%), and the UK (1981; 10.4%).

**Table 1:** The top 10 countries of origin of papers in novel coronavirus (COVID-19) research

Ranking	Country	Number of publications (%)
1st	United States	4479 (23.5)
2nd	China	3310 (17.4)
3rd	Italy	2314 (12.2)
4th	United Kingdom	1981 (10.4)
5th	India	1104 (5.8)
6th	France	881 (4.6)
7th	Canada	790 (4.1)
8th	Germany	742 (3.9)
9th	Spain	680 (3.6)
10th	Australia	676 (3.5)

During the early stage of the COVID-19 outbreak, a total of 8387 institutions were identified. The top 10 institutions that published the most publications on COVID-19 were shown in Table 2. The *Huazhong University of Science and Technology* was the most productive institution with 422 publications, followed by *Tongji Medical College* with 415 publications, and *Harvard Medical School* with 331 publications.

**Table 2:** The top 10 institutions contributed to publications on novel coronavirus (COVID-19) research

Ranking	Institution	Country	Number of publication (%)
1st	*Huazhong University of Science and Technology*	China	422 (2.22)
2nd	*Tongji Medical College*	China	415 (2.18)
3rd	*Harvard Medical School*	USA	331 (1.74)
4th	*Inserm (French National Institute of Health and Medical Research)*	France	272 (1.43)
5th	*Università degli Studi di Milano*	Italy	258 (1.35)
6th	*University College London*	UK	237 (1.24)
7th	*Università degli Studi di Roma La Sapienza*	Italy	232 (1.22)
8th	*IRCCS Foundation Rome*	Italy	223 (1.17)
9th	*University of Toronto*	Canada	210 (1.10)
10th	*University of Oxford*	UK	191 (1.00)

Amongst the top 10 journals shown in Table 3. *British Medical Journal* with IF, 2019 = 30.223, published the most number of publications on COVID-19 (*n* = 522), followed by *Journal of Medical Virology* (*n =* 311; IF, 2019 = 2.021), *Lancet* (*n* = 215; IF, 2019 = 60.392), and *Journal of the American Medical Association* (*n* = 137; IF, 2019 = 45.540).

**Table 3:** The top 10 journals that published articles on novel coronavirus (COVID-19) research

Ranking	Journal	Number of documents	IF ^**a**^
1st	*British Medical Journal*	522 (2.74)	30.223
2nd	*Journal of Medical Virology*	311 (1.63)	2.021
3rd	*Lancet*	215 (1.13)	60.392
4th	*Journal of the American Medical Association*	137 (0.72)	45.540
5th	*Journal of Infection*	135 (0.71)	4.842
6th	*International Journal of Environmental Research and Public Health*	131 (0.69)	2.849
7th	*Medical Hypotheses*	129 (0.68)	1.375
8th	*Lancet Infectious Diseases*	126 (0.66)	24.446
9th	*International Journal of Infectious Diseases*	125 (0.66)	3.202
10th	*Infection Control and Hospital Epidemiology*	122 (0.64)	2.938

^a^ Impact factors (IF) based on Clarivate Analytics ‘Journal Citation Reports (JCR) 2019 which was published in 2020

**Fig. 1:**
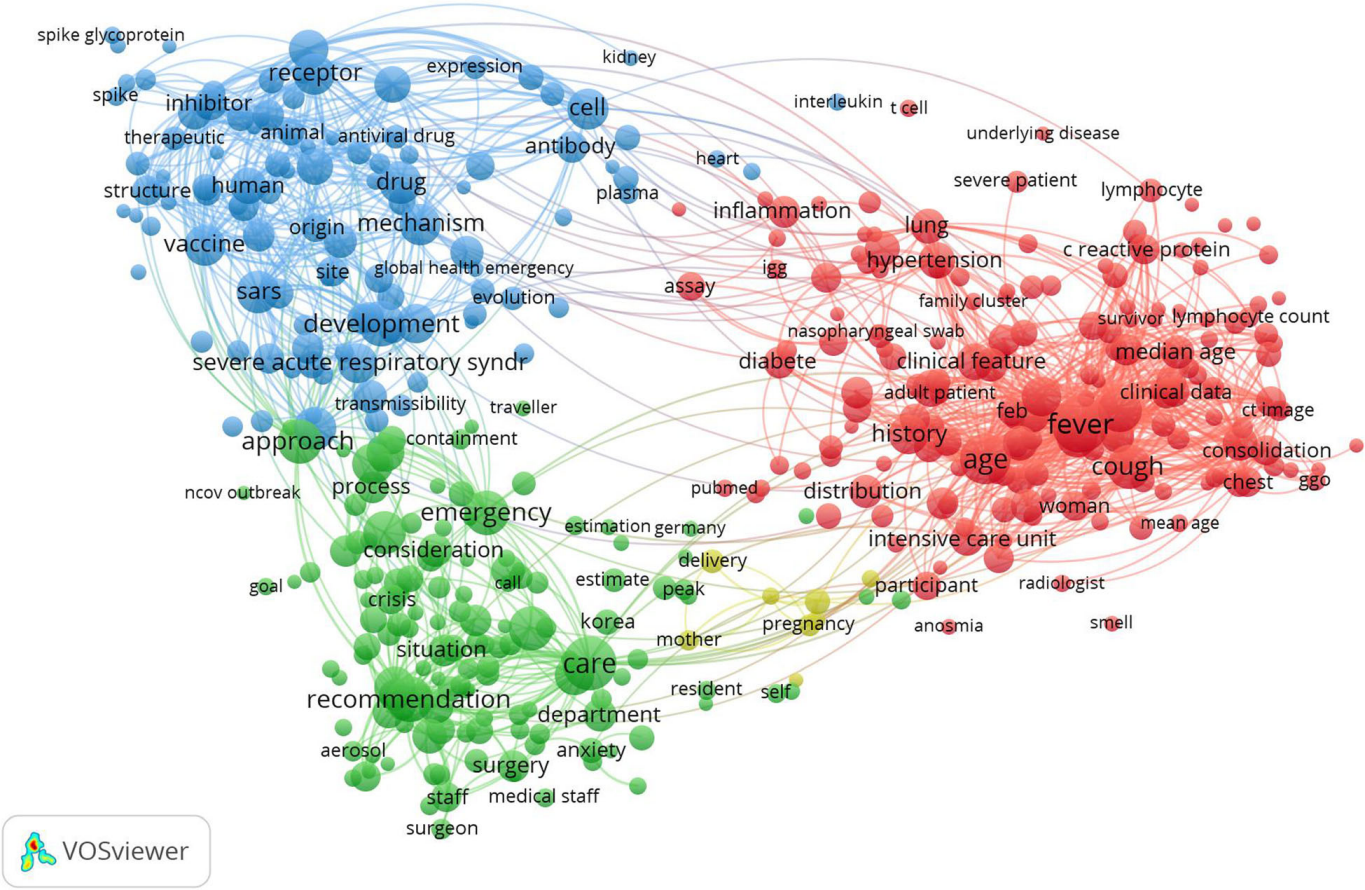
Research topics clustered by mapping of co-occurrences of terms in title/abstract for publications related to COVID-19. Of the 20,897 terms, 721 terms have occurred at least 10 times. For each of the 721 terms, a relevance score was determined and used to select the 60% most relevant terms. The size of the circles in Fig. 1 represents the occurrences of terms in title/abstract. The largest set of connected terms consists of 433 terms in four clusters: Clinical features studies (red), pathological findings and therapeutic design (blue), care facilities preparation and infection control (green), and maternal, perinatal and neonatal outcomes (yellow)

Research hot topics for publications related to COVID-19 were visualized and presented in network visualization by mapping of co-occurrences of terms in title/abstract for the top-2000 most cited publications (Fig. 1). Of the 20,897 terms, 721 terms occurred at least 10 times. The largest network of connected terms involves of 433 terms in four clusters. The four most used topics in publications related to COVID-19 are signified by four colored clusters: red, blue, green, and yellow colors. Cluster number 1 (red color) involved terms related to clinical features and characteristics topic such as “fever”, “cough”, “severe patients”, “diabetes”, “hypertension” or “C-reactive protein”; Cluster number 2 (blue color) involved terms related to pathological findings and therapeutic design topic such as “receptor”, “enzyme”, “inhibitor”, “angiotensin”, “spike glycoprotein”, “drug”, “antiviral” or “chloroquine”; Cluster number 3 (green color) involved terms related to care facilities preparation and infection control topic such as “control measures”, “recommendations”, “preparedness”, “experience” or “medical staff”; and Cluster number 4 (yellow color) involved terms related to maternal, perinatal and neonatal outcomes topic such as “delivery”, “infant”, “mother”, “neonate”, or “newborn”.

The citation counts for the final 20 articles ranged from 387 to 2554 (Table 4). All documents were published in 7 different journals [3, 7, 9, 46–62]. Most documents were published in *New England Journal of Medicine* (*n* = 7), followed by the *Lancet* (*n* = 6), *Lancet Respiratory Medicine* (*n* = 2), *Journal of the American Medical Association* (*n =* 2), *Cell Research* (*n* = 1), *Nature* (*n =* 1), and *Cell* (*n =* 1).

**Table 4:** The Top 20 Cited Papers in novel coronavirus (COVID-19) research

Ranking	Authors	Title	Year	Source title	Cited by
1st	Huang et al. [7]	“Clinical features of patients infected with 2019 novel coronavirus in Wuhan, China”	2020	*The Lancet*	2554
2nd	Wang et al. [9]	“Clinical Characteristics of 138 Hospitalized Patients with 2019 Novel Coronavirus-Infected Pneumonia in Wuhan, China”	2020	*Journal of the American Medical Association*	1507
3rd	Guan et al. [46]	“Clinical characteristics of coronavirus disease 2019 in China”	2020	*New England Journal of Medicine*	1469
4th	Zhu et al. [47]	“A novel coronavirus from patients with pneumonia in China, 2019″	2020	*New England Journal of Medicine*	1393
5th	Chen et al. [48]	“Epidemiological and clinical characteristics of 99 cases of 2019 novel coronavirus pneumonia in Wuhan, China: a descriptive study”	2020	*The Lancet*	1322
6th	Li et al. [49]	“Early transmission dynamics in Wuhan, China, of novel coronavirus-infected pneumonia”	2020	*New England Journal of Medicine*	1061
7th	Zhou et al. [3]	“Clinical course and risk factors for mortality of adult inpatients with COVID-19 in Wuhan, China: a retrospective cohort study”	2020	*The Lancet*	980
8th	Wu and McGoogan [50]	“Characteristics of and Important Lessons from the Coronavirus Disease 2019 (COVID-19) Outbreak in China: Summary of a Report of 72,314 Cases from the Chinese Center for Disease Control and Prevention”	2020	*Journal of the American Medical Association*	964
9th	Zhou et al. [51]	“A pneumonia outbreak associated with a new coronavirus of probable bat origin”	2020	*Nature*	931
10th	Chan et al. [52]	“A familial cluster of pneumonia associated with the 2019 novel coronavirus indicating person-to-person transmission: a study of a family cluster”	2020	*The Lancet*	805
11th	Lu et al. [53]	“Genomic characterisation and epidemiology of 2019 novel coronavirus: implications for virus origins and receptor binding”	2020	*The Lancet*	724
12th	Holshue et al. [54]	“First case of 2019 novel coronavirus in the United States”	2020	*New England Journal of Medicine*	585
13th	Yang et al. [55]	“Clinical course and outcomes of critically ill patients with SARS-CoV-2 pneumonia in Wuhan, China: a single-centered, retrospective, observational study”	2020	*The Lancet Respiratory Medicine*	521
14th	Wang et al. [56]	“Remdesivir and chloroquine effectively inhibit the recently emerged novel coronavirus (2019-nCoV) in vitro”	2020	*Cell Research*	507
15th	Xu et al. [57]	“Pathological findings of COVID-19 associated with acute respiratory distress syndrome”	2020	*The Lancet Respiratory Medicine*	485
16th	Van Doremalen et al. [58]	“Aerosol and surface stability of SARS-CoV-2 as compared with SARS-CoV-1″	2020	*New England Journal of Medicine*	470
17th	Hoffmann et al. [59]	“SARS-CoV-2 Cell Entry Depends on ACE2 and TMPRSS2 and Is Blocked by a Clinically Proven Protease Inhibitor”	2020	*Cell*	463
18th	Rothe et al. [60]	“Transmission of 2019-NCOV infection from an asymptomatic contact in Germany”	2020	*New England Journal of Medicine*	403
19th	Mehta et al. [61]	“COVID-19: consider cytokine storm syndromes and immunosuppression”	2020	*The Lancet*	389
20th	Zou et al. [62]	“SARS-CoV-2 viral load in upper respiratory specimens of infected patients”	2020	*New England Journal of Medicine*	387

## Discussion

The purpose of this bibliometric study was to summarize and examine the evolution of the immediate effect of the COVID-19 pandemic on scientific output. The findings of the study reflect the latest global scholarly publications on COVID-19. The analysis of this study showed some significant insights. The current study has shown a rapid increase in research activities related to COVID-19 over such a short period of time compared to other diseases or infections [14–16, 18, 21, 22, 63–66]. This rapid increase in research output on COVID-19 in such a short period of time is due to several reasons: COVID-19 is a global pandemic that has impacted and influenced the global health status, due to a lockout in many countries where scientists have more time to write and publish, and most of the journals considered COVID-19 related papers as a top priority for publication and their editorial process is fast-tracked [31].

The current study has revealed the leading role played by the USA, China, Italy, and the UK, in COVID-19 research. A potential reason for these findings may be attributed to the high prevalence of COVID-19 in those countries witnessing the first outbreak [67–71]. The USA tends to have superior conditions for basic medical research or experimental trials, including sufficient funding and resources, advanced equipment, and skilled researchers [34].

As we have seen in our evidence maps on the main topics, a large number of articles focused on clinical features studies, pathological findings and therapeutic design, care facilities preparation and infection control, and maternal, perinatal and neonatal outcomes Meanwhile, all these topics have been emerging commonly in recent months and may become a major topic in the next years, particularly after COVID-19 in Wuhan as suggested by a more recent study [33].

The current study showed that most of the top-cited articles were published in high impact journals. Scientists are likely to rely on these Journals for higher impact [72]. Many journals, including all leading journals with high impact factors, highlighted specific issues of COVID-19 and most publishers published them as a top priority for their publication and also provided free access to such papers [31].

In the current study, highly cited articles were evidence-based research, for example, the first most cited article was from Huang et al. [7] in the *Lancet.* This article focused on the epidemiology, laboratory diagnosis, sign and symptoms, and clinical outcomes of 41 patients who were reported as having COVID-19 infection. In addition, this study [7] demonstrated that COVID-19 infection caused serious respiratory disease clusters and was linked to ICU mortality. The second most cited study was from Wang et al. [9] in the *Journal of the American Medical Association.* The aim of this study was to describe the clinical characteristics of patients with COVID-19-infected pneumonia in Wuhan, China. The third most cited was from Guan et al. [46] in the *New England Journal of Medicine.* This study aimed to describe the clinical features of Covid-19 in a selected cohort of patients across China. The fourth most cited study was from Zhu et al. [47] in the *New England Journal of Medicine.* The purpose of this study was to characterize a novel coronavirus found in patients with pneumonia and to identify the source of the pneumonia clusters whose specimens were tested by the China CDC at an early stage of the outbreak.

### Strengths and limitations

Bibliometric and visual analysis has been performed to represent the current status of COVID-19 research through analysis of citation patterns and hot topics in this field. This provides quick information during the early stage of the outbreak that shows important patterns in several different dimensions, which to the best of our knowledge is the first analysis of its type in the field. A limitation of our study was that only the Scopus database was used for article retrieval. Other databases, like PubMed, were not considered. The total number of publications related to COVID-19 from PubMed could be a little bit higher than Scopus. PubMed is updated daily, including online in an early version by various journals. In contrast, Scopus is readily updated for published issues but does not include the online version of publications before inclusion in an issue for most indexed journals [37]. Although several databases are used in bibliometric studies at the global level [37, 38, 73], our study applied the Scopus database for data extraction, which is commonly accepted by investigators for high-quality bibliometric analysis [74–80]. Furthermore, Scopus contains a higher degree of features than PubMed, including the affiliations for all authors and citations per document [38, 81]. In addition, it should be noted the limitation of the speed at which evidence appears, which undoubtedly influences the actuality of the manuscript. Therefore, we emphasized that this bibliometric analysis only represents the initial phase of the pandemic. Thus, studies published in Scopus after June 19, 2020, were not included in this study.

## Conclusions

This bibliometric study may reflect rapidly emerging topics on COVID-19 research, where substantial research activity has already begun extensively during the early stage of the outbreak. Overall, our results may provide useful information to outline new viewpoints and shape future directions for COVID-19 research. COVID-19 research is a hot issue nowadays. Clinical features studies, pathological findings and therapeutic design, care facilities preparation and infection control, and maternal, perinatal and neonatal outcomes could be a research frontier in the future.

## Data Availability

The datasets generated and/or analysed during the current study are available upon request to the corresponding authors (saedzyoud@yahoo.com; samahjabi@yahoo.com).

## Abbreviations

COVID-19: Coronavirus disease 2019
JCR: Journal Citation Reports;
IFs: Impact factors
2019-nCoV: 2019 novel coronavirus.
